# Venereal Buboes

**Published:** 1897-03

**Authors:** 


### VENEREAL BUBOES.

Dr. J. C. Perry (*American Journal of the Medical Sciences*) has employed the treatment described by Dr. Hayden in seven cases with extremely satisfactory results. This method consists in making a small puncture of the skin at the most fluctuating point of the bubo; thorough evacuation of the pus; irrigation of the cavity with peroxide of hydrogen, followed by bichloride solution 1:1000; removal of all fluid by compression, and distention of the cavity with warm vaseline holding in suspension 10 per cent. iodoform; and application of a cold bichloride dressing to favor congealment. Of Dr. Perry's patients five were cured by one injection, the average time of treatment being thirteen and one-half days. On the ground of his experience he concludes:

1. That buboes are probably caused by the absorption of chemical poisons, the result of the action of the micro-organisms in the chancroid, and not to the entrance of the micro-organisms themselves into the lymphatics.

2. That the benzoate of mercury yields such satisfactory results that it should be employed in the treatment of non-suppurating buboes, and excision reserved for those cases in which benzoate has failed.

3. The injection of iodoform ointment should be used in the treatment of all freely suppurating buboes, since statistics show that it yields much more satisfactory results than the other methods of treatment applicable to this variety.

4. Incision and curettement should be used in a few cases in which the skin has been destroyed and the ulcer presents an unhealthy granulating surface.

5. Excision should be reserved for cases that have not yielded to other treatment, and for those in which there are several foci of suppuration.

